# Immunological Drug–Drug Interactions Affect the Efficacy and Safety of Immune Checkpoint Inhibitor Therapies

**DOI:** 10.1021/acs.chemrestox.4c00067

**Published:** 2024-06-24

**Authors:** Sophie Grice, Anna Olsson-Brown, Dean J. Naisbitt, Sean Hammond

**Affiliations:** † Department of Molecular and Clinical Pharmacology, Institute of Translational Medicine, University of Liverpool, Liverpool L69 3GE, U.K.; ‡ Sussex Cancer Centre, University Hospitals Sussex, Brighton BN2 5BD, U.K.; § ApconiX, Alderley Edge SK10 4TG, U.K.

## Abstract

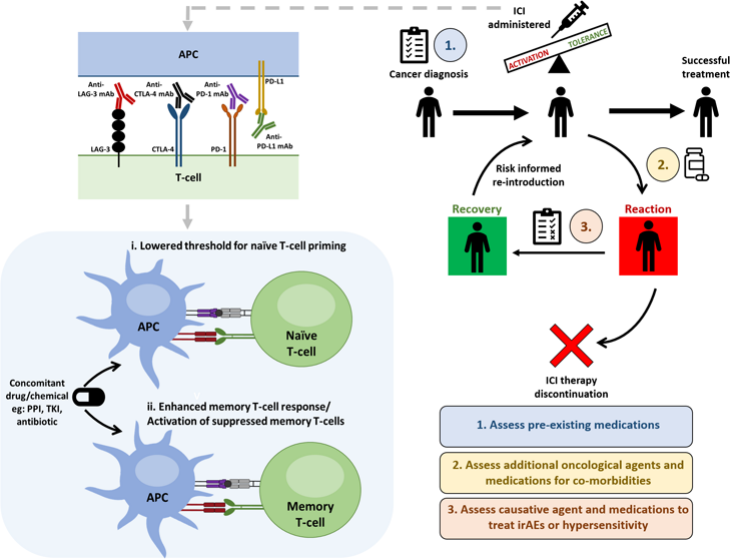

With the rapid expansion in the development and clinical utility of immune checkpoint inhibitors (ICIs) for oncology, the continual evaluation of the safety profile of such agents is imperative. The safety profile of ICIs as monotherapy is dominated by immune-related adverse events, which can be considered as an extension of the mechanism of action of these immunomodulatory drugs. Further to this, an emerging theme is that ICI treatment can significantly impact upon the tolerability of coadministered medications. Numerous reports in literature indicate that ICIs may alter the immunological perception of coadministered drugs, resulting in undesirable reactions to a variety of concomitant medications. These reactions can be severe in manifestation, including hepatotoxicity and Stevens-Johnson Syndrome (SJS)/toxic epidermal necrolysis (TEN), but may also have detrimental impact on malignancy control. To minimize the impact of such drug–drug interactions on patients, it is imperative to identify medications that may cause these reactions, understand the underlying mechanisms, consider the timing and dosing of comedication, and explore alternative medications with comparable efficacies. Improving our understanding of how concomitant medications affect the safety and efficacy of ICIs can allow for potential culprit drugs to be identified/removed/desensitized. This approach will allow the continuation of ICI therapy that may have been discontinued otherwise, thereby improving malignant control and patient and drug development outcomes.

## Introduction

T-cells are known to play a critically important role in immunosurveillance and clearance of tumors.^1^ Simply described, the central dogma of αβ T-cell mediated immunity is a three-signal model: Signal 1 is generated after antigens in the form of peptides derived from an endogenously or exogenously sourced protein are presented to the T-cell receptor via major histocompatibility complexes (MHC). The interpretation of this signal and subsequent downstream response of the T-cell is then determined by additional signaling; signal 2: actions of costimulatory and immune checkpoint/coinhibitory pathways; and signal 3: cytokine signaling. For over a decade, evasion of immune detection and destruction has been recognized as one of the hallmarks of cancer.^2^ Therefore, it is no surprise that tumor cells regularly evolve to avoid their amenability to immunosurveillance through subversion of all three of these signals.^3^ In particular, tumor cells often perturb signal 2 through the expression of ligands for immune checkpoints on their cell surface, thereby disrupting antitumor T-cell responses.^4^ Immune checkpoints are involved in multiple immune regulation pathways in multiple parts of the body.

The immune checkpoint cytotoxic T-lymphocyte-associated protein-4 (CTLA-4) is expressed on CD4+ (activated/exhausted, Tregs), CD8+ (activated/exhausted), and some tumor cells and competes with the costimulatory receptor CD28 for binding to their ligands CD80 or CD86, which are found on antigen presenting cells, thereby inhibiting T-cell activation.^5^ Programmed cell death protein 1 (PD-1) is an additional immune checkpoint expressed on CD4+ T-cells (activated/exhausted, follicular), CD8+ T-cells (activated/exhausted), B cells, dendritic cells, monocytes, mast cells, and Langerhans cells.^5,6^ PD-1 inhibits T-cell activation by interacting with its ligands programmed death-ligand 1 (PD-L1) or PD-L2 found on antigen presenting cells, CD4+ T-cells, nonlymphoid tissues, and some tumor cells.^5,6^ Lymphocyte activation gene-3 (LAG-3) expressed on CD4+ T-cells (Treg and exhausted), CD8+ T-cells (exhausted), and natural killer cells (NK) also plays a significant role in regulating T-cell activation by binding to MHC class II molecules on antigen presenting cells, liver cells, and some tumor cells.^5,7^ The authors refer readers to refs (5), (8), and (9) for discussion of immune regulation pathways. Therapeutic manipulation of signal 2 has been exploited to great effect in recent years. The emergence of immune checkpoint inhibitors (ICIs) as a therapeutic option has dramatically altered the landscape of oncological treatments, leading to the emergence of long-term and enduring years-long malignant control for a variety of cancer indications (Table 1 and Figure 2). The marketed class of ICIs considered here are monoclonal antibodies (mAbs) which target PD-1, CTLA-4, and LAG-3 coinhibitory pathways and are often administered with great efficacy as monotherapy, as combinational therapy, or in combination with other oncological agents.^10,11^

**Figure 1:**
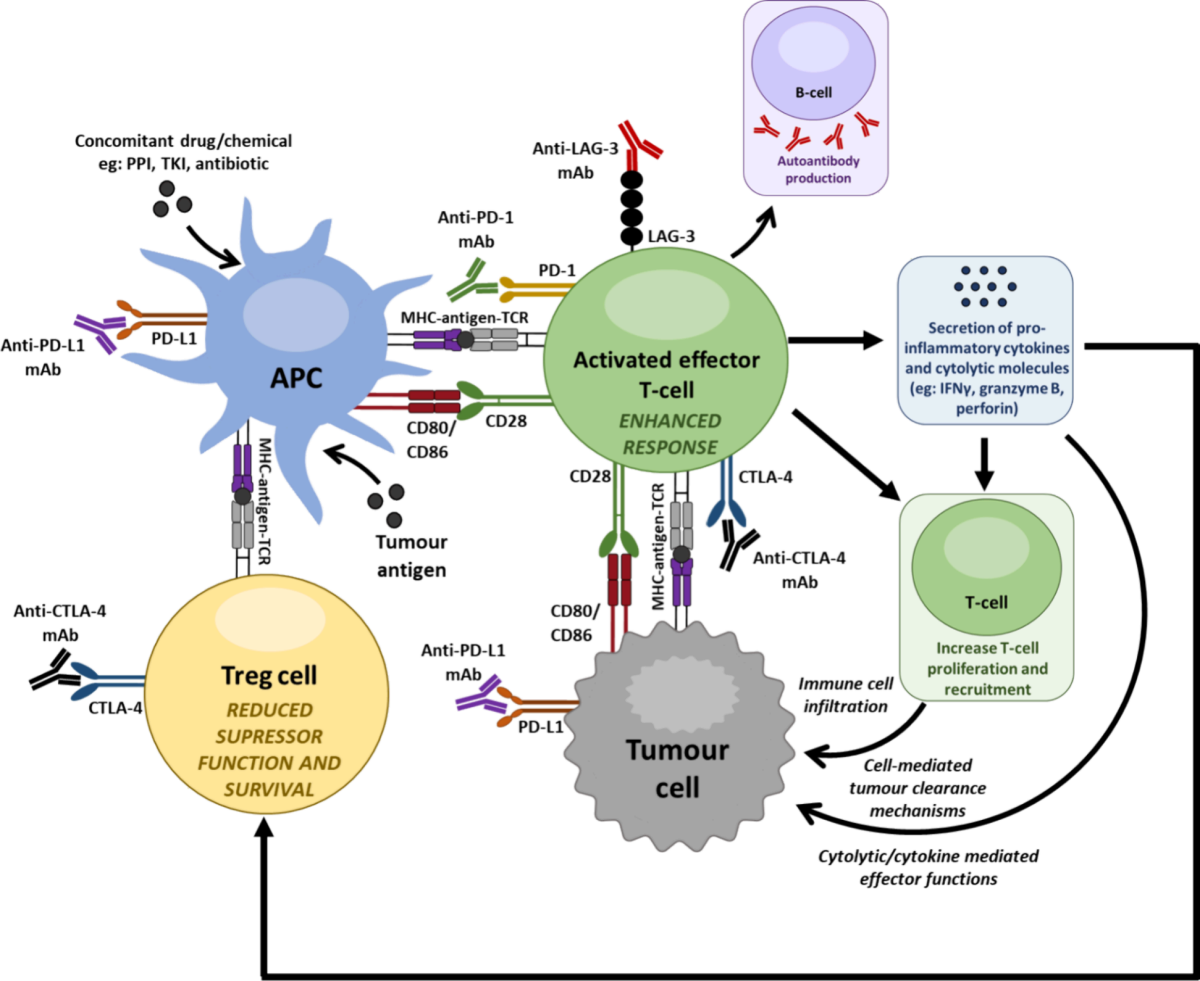
Overview of aspects of signal 2 and how immune checkpoint inhibitor agents target coinhibitory receptors CTLA-4/PD-1/LAG-3 or ligand PD-L1 enabling the activation of immune response by costimulatory receptors binding to their ligands subsequently resulting in antitumor responses. Tumor antigens as well as drugs/chemicals administered during ICI therapy can be presented on antigen presenting cells (APC) resulting in similar T-cell responses including increased T-cell proliferation and recruitment, secretion of proinflammatory cytokines and cytolytic molecules, production of autoantibodies, and reduction in Treg cell suppressor functions and survival. PPI: proton pump inhibitor, TKI: tyrosine kinase inhibitor, mAb: monoclonal antibody, MHC: major histocompatibility complex, TCR: T-cell receptor, PD-1: programmed death protein 1, PD-L1: programmed death-ligand 1, CTLA-4: cytotoxic T-lymphocyte-associated protein-4, LAG-3: lymphocyte activation gene-3 protein.

**Table 1:** Immune Checkpoint Inhibitors Approved in Europe and the United States^a^

**Immune checkpoint inhibitor**	**Commercial name**	**Backbone**	**Light chain**	**Target for inhibition**	**Date of European approval**	**Date of United States approval**
Ipilimumab	Yervoy	IgG1	Kappa	CTLA-4	2011	2011
Nivolumab	Opdivo	IgG4k	Kappa	PD-1	2015	2014
Pembrolizumab	Keytruda	IgG4	Kappa	PD-1	2015	2014
Atezolizumab	Tecentriq	IgG1	Kappa	PD-L1	2017	2016
Avelumab	Bavencio	IgG1	Lambda	PD-L1	2017	2017
Durvalumab	Imfinzi	IgG1	Kappa	PD-L1	2018	2017
Cemiplimab	Libtayo	IgG4	Kappa	PD-1	2019	2018
Dostarlimab	Jemperli	IgG4	Kappa	PD-1	2021	2021
Relatlimab	Opdualag (relatlimab + nivolumab combo)	IgG4	Kappa	LAG-3	2022	2022
Tremelimumab	Imjudo	IgG2	Kappa	CTLA-4	2023	2022
Retifanlimab	Zynyz	IgG4	Kappa	PD-1	2023	2023

a PD-1: programmed death protein 1, PD-L1: programmed death-ligand 1, CTLA-4: cytotoxic T-lymphocyte-associated protein-4, LAG-3: lymphocyte activation gene-3 protein.

Unfortunately, the on-target pharmacology of these agents is not restricted to tumor tissue. Indeed, ICIs are known to work at multiple sites (e.g., secondary lymphoid tissue and level of tumor). As such, the systemic deregulation imposed by these agents is accompanied by what is now a widely recognized toxicity profile of on-target off-tumor pharmacology known as immune-related adverse events (irAEs). The etiology of these toxicities is therefore the initiation and propagation of aberrant immune responses to xenobiotics and self-antigens.^9,12,13^ Up until now, the elucidation of a given patient’s propensity for irAEs, the identity of the target antigens, and biomarkers for accurate prediction and detection of such reactions remain key challenges in the clinical arena of immune-oncology (IO) therapy. In recent times, it has become apparent that one class of relevant xenobiotic agents (therapeutic drugs) may be responsible for an underappreciated portion of such reactions. The principal focus of this review is therefore to discuss the experimental and clinical burden of proof for what appears to be a suboptimally managed class of immunological drug–drug interactions that impact the safety and efficacy profile of IO agents in real-world clinical practice.

## Mechanisms of irAEs and Overlap with Drug Hypersensitivity

Drug hypersensitivity classically refers to an adverse drug reaction (ADR) of immune etiology, which occurs when an individual is exposed to a drug generally tolerated by others.^14^ Mechanisms of antigenic stimulation of T-cells include the hapten and prohapten mechanism, pharmacological interaction (PI) mechanism, and altered peptide repertoire mechanism. The hapten and prohapten concept proposes that drugs and metabolites can bind covalently to proteins, forming hapten–protein complexes. These are then processed by antigen presenting cells into drug or metabolite peptide fragments which are presented on the cell surface by MHC to the T-cell receptor. The PI mechanism does not require antigen processing, drugs and metabolites can bind directly, noncovalently, and reversibly to MHC proteins or peptides embedded in the MHC peptide binding cleft resulting in T-cell activation. Finally, the altered peptide repertoire mechanism occurs when a drug can bind within the MHC binding cleft altering the repertoire of presented endogenous peptides, this has only been described for abacavir to date. The authors refer readers to the following reviews for further discussion on general mechanisms of drug hypersensitivity.^15,16^

**Figure 2:**
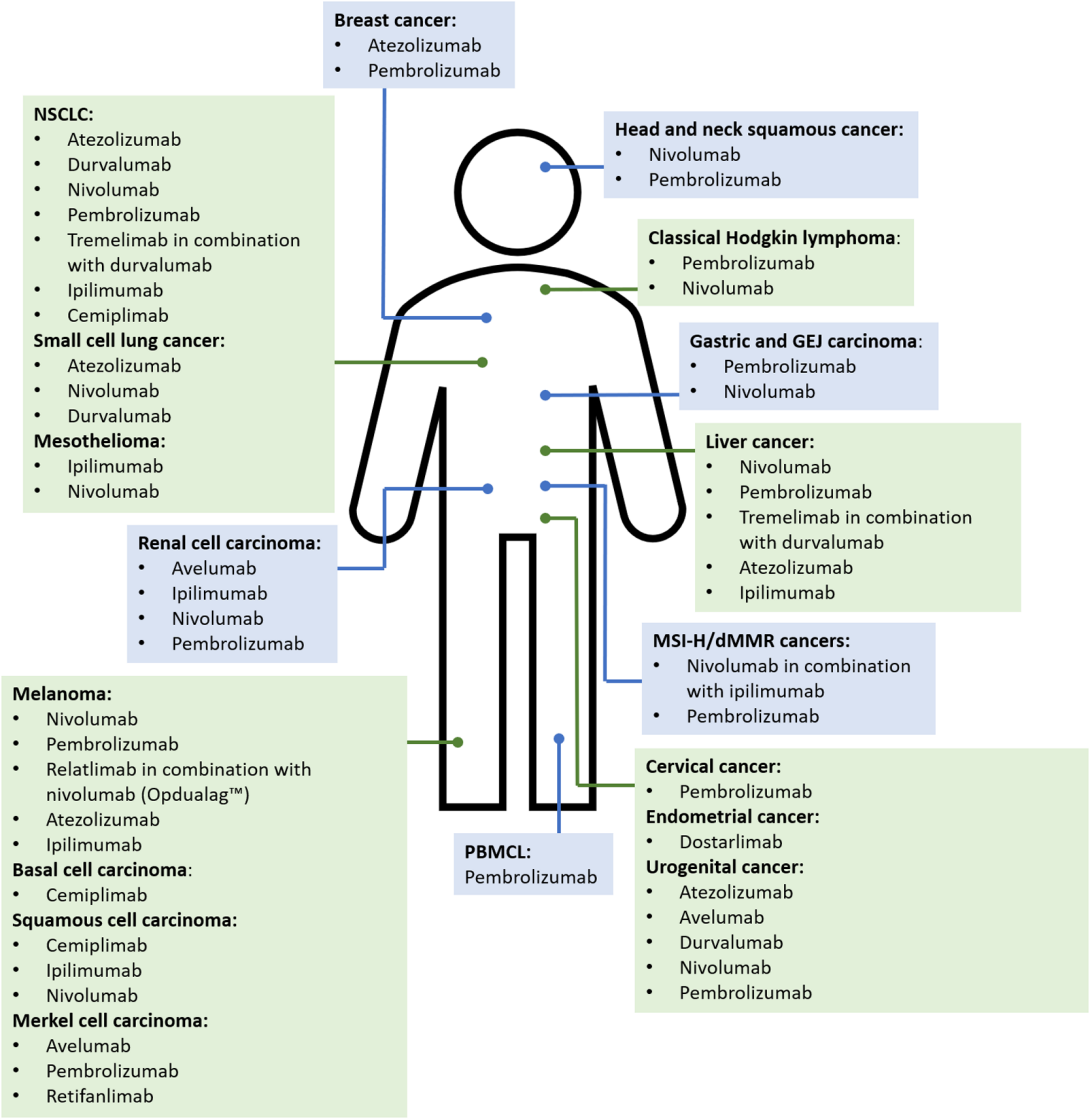
Cancer types and immune checkpoint inhibitor therapies that are approved for each cancer type. NSCLC: non-small cell lung cancer, MSI-H: microsatellite instability-high, dMMR: mismatch repair deficient cancer, GEJ: gas-tresophageal junction, PBMCL: primary mediastinal large B-cell lymphoma.

**Figure 3:**
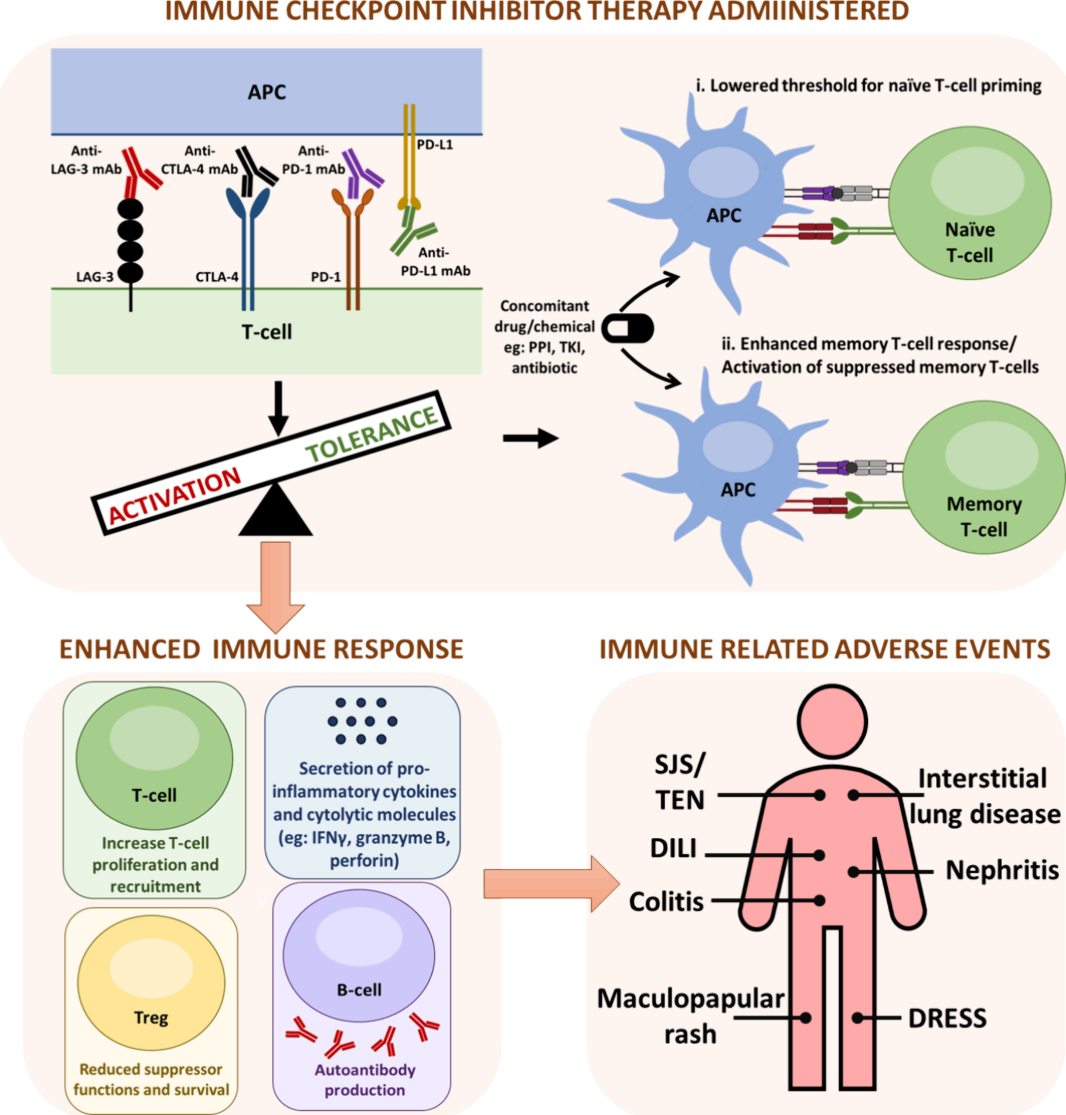
Overview of how the administration of immune checkpoint inhibitors can lead to i. lowered threshold for naïve T-cell priming to concomitantly administered medications or ii. enhanced memory T-cell responses or activation of suppressed drug specific memory T-cell responses to concomitantly administered medications. The enhanced T-cell mediated immune response leads to increases in T-cell proliferation and recruitment, secretion of proinflammatory cytokines and cytolytic molecules, production of autoantibodies, and decreases in Treg cell function and survival, which can result in immune-related adverse events. SJS: Stevens-Johnson syndrome, TEN: toxic epidermal necrolysis, DRESS: drug reaction with eosinophilia and systemic symptoms syndrome.

As the IO field has developed, the frequency of use of ICIs for the treatment of numerous cancers is increasing; ICI treatments are being used earlier in the oncology pathway as neoadjuvant and adjuvant therapies. The mechanism of action of marketed ICIs is via blockade of coinhibitory signaling pathways mediated by PD-1, CTLA-4, and LAG-3. This leads to the enhancement of antitumor efficacy through the alleviation of negative regulation. However, by the same token, this widespread removal of the “immunological brakes” also results in aberrant deployment of the adaptive immune system against nontumor or nontumor specific antigens, sometimes with destructive consequences. These adverse reactions known as irAEs are as heterogeneous in presentation as the antigens they focalize on: they therefore have the potential to affect all organ systems.^17^ Mechanistically, irAEs can be the result of enhanced pre-existing responses, *de novo* adaptive responses, cross-reactivity of antigens, depletion of tolerance, tissue microenvironment polarization, and combinations thereof. Across all grades, irAEs occur in up to 80% of ICI-treated patients manifesting as endocrine, gut, lung, neurological, musculoskeletal, and skin toxicities; therefore, understanding the mechanisms of these irAEs is crucial in optimizing ICI patient safety profiles (Figures 1 and 3). For a detailed review of the heterogeneous manifestations of irAEs following ICI administration, the authors refer readers to several reviews.^9,12,13,18^

Over the past decade, several warnings from research indicate that the inhibition of immune regulation may exacerbate ADRs *in vitro* and *in vivo*. Sulfamethoxazole is an antibiotic that is metabolized to reactive oxidative metabolites nitroso-sulfamethoxazole (SMX-NO) and sulfamethoxazole-hydroxylamine. Sulfamethoxazole is associated with a high number of hypersensitivity reactions; research stipulates that SMX-NO acts as a hapten, binding covalently to proteins before being processed by antigen presenting cells where a peptide–MHC complex is formed which is presented to the T-cell receptor.^19^ SMX-NO is now commonly used in T-cell-based assays as a model antigen. During *in vitro* T-cell priming experiments to SMX-NO and epigallocatechin gallate (catechin of green tea), the blockade of immune checkpoints using anti-PD-1, PD-L1, and CTLA-4 mAbs significantly lowered the threshold for the priming of naïve T-cells.^20−24^ In addition to influencing naïve T-cell priming, there is evidence that the *in vitro* and *in vivo* administration of ICIs also influences the threshold at which previously suppressed memory T-cells expand and become effector T-cells. Lymphocyte transformation tests (LTTs) were carried out using peripheral blood mononuclear cells (PBMC) from healthy donors and ICI-treated patients before and 1 week after their ICI therapy; the *in vitro* (healthy donors) and *in vivo* (ICI patients) administration of ICIs caused a significant increase in PBMC proliferation in response to the re-exposure of Bandrowski’s base (chemical found in hair dye).^22^ Additionally, Sugita et al. reported a patient with suspected nickel contact dermatitis. Their initial LTT was negative; however, with the addition of anti-CTLA-4 mAb *in vitro*, the patient LTT was positive to nickel thereby improving the sensitivity of the LTT.^25^ These studies indicate that the addition of ICIs allows for an enhancement of the expansion and functionality of memory T-cells.

*In vivo* studies also supported this risk. In 2015, idiosyncratic drug-induced liver injury (DILI) was modeled using the combination of PD-1 knockout mice treated with an anti-CTLA-4 antibody; this immune checkpoint combination was able to unmask liver injury caused by amodiaquine.^26^ Similar findings were described in this model with isoniazid and nevirapine.^27^ When mice were treated with drug alone, liver injury was insignificant, yet with the inhibition of immune tolerance mechanisms, the liver injury observed in the mice was significantly increased, particularly when multiple methods of inhibition were carried out. Similar enhancements in liver injury caused by epigallocatechin gallate were seen in the model.^28^ These studies provided proof of principle that the inhibition of immune checkpoints could increase the potential for comedications to incite irAEs such as DILI.

The concept of an immunological drug–drug interaction is not unprecedented. In fact, early clinical recognition of this type of interaction can be found with an older form of cancer immunotherapy: high-dose IL-2 therapy. The increased incidence of delayed hypersensitivity reactions observed with patients treated with high-dose IL-2 who were exposed to radiocontrast media in short succession was so prevalent that it is cited on early and subsequent iterations of the FDA label for PROLEUKlN.^29,30^ Literature cited to support this interaction originates ca. 1990s,^31,32^ though the nature of the reactions outlined may not always reflect true adaptive hypersensitivity. Recently, a large prospective study has provided a renewed and timely revistation of this interaction^33^ again; though not conclusive in mechanistic divulgence, a clear impact on the safety profile of the contrast media by concomitant IL-2 treatment is observed. As ICIs continue to take on and advance the mantle once firmly held by immune-cytokine therapy, it is apparent that they have brought about a renaissance of this particular toxicity.

## Medications to Ameliorate Immune-Related Adverse Events (irAEs)

The most reported ICI-induced irAEs include rash, fatigue, colitis, muscle pain/weakness, and pneumonitis.^34^ ICIs can also cause cardiovascular adverse events, particularly ICI-associated myocarditis, which can occur in ∼5% of the patient population.^34^ This is a severe adverse reaction, which is fatal for 27–60% of patients who have this toxicity, with the fatality rate increasing with combinational ICI therapy and ICI therapy in combination with tyrosine kinase inhibitors (TKIs).^35,36^ The CTLA-4 inhibitor ipilimumab tends to cause a greater incidence of higher-grade adverse events than PD-1 or PD-L1 inhibitors; this may be due to its additional effect on the depletion of Treg cells.^37^ Combinational ICI therapy is often associated with better overall outcomes for a variety of cancer indications; notably, in the landmark checkmate 067 study, previously untreated metastatic melanoma patients had a higher progression free survival of 11.5 months when administered nivolumab plus ipilimumab compared to 6.9 months with nivolumab monotherapy and 2.8 months with ipilimumab monotherapy.^3,10,38,39^ This may be due to synergistic effects of relieving effector T-cells from PD-1/PD-L1 mediated anergy and the depletion of intratumoral Tregs.^37^ This is however accompanied by increased incidence of irAEs. Larkin et al. reported treatment for grade 3 or 4 toxicity occurred in 55.0% of those in the nivolumab plus ipilimumab group, 16.3% of the patients in the nivolumab monotherapy group, and 27.3% of those in the ipilimumab monotherapy group.^10^ The incidence of irAEs provides evidence that immune dysregulation has occurred in patients; however, it may not directly be representative of efficacy. It must be noted that toxicity can occur without efficacy and vice versa due to the malignant nonspecific nature of the mechanism of action of ICIs. These irAEs can be managed therapeutically, typically by the administration of corticosteroids and other immunosuppressive agents. Given that irAEs often resemble autoimmune conditions, there has been a clinical precedence that treatment algorithms for autoimmune diseases be applied to corresponding tissue-specific irAEs. However, given the mechanism of irAEs and the novel clinical manifestations, these algorithms are not optimized for treatment of these toxicities, so prospective, mechanistically focused trials are required. Some novel alternative treatments including faecal microbiota transplantation for the treatment of ICI-induced colitis are emerging, which has been implemented with considerable success rates.^40^ Additionally, questions over the earlier use of T-cell and cytokine directed therapies are arising, as the incidence of irAEs rises in line with increased usage and more patients are requiring treatment.

### Corticosteroids

The most frequently administered pharmaceuticals for the treatment of ICI-associated adverse events are glucocorticoid steroids. It was reported that 38% of 412 advanced melanoma patients who received ICI therapy required glucocorticoids to treat toxicities.^41^ Prolonged use of corticosteroids is associated with adverse side effects such as insulin resistance, altered mental health, osteoporosis, and increased risk of infections.^42^ Perhaps the most important consideration is whether the immunosuppressive nature of corticosteroids counteracts the antitumor effects of ICIs themselves. This is a contested subject, and several analyses have investigated this topic, showing differing results likely due to the highly biased, inconsistent nature of retrospective data with differing real-world patient management. Interestingly, in terms of irAEs closely related to efficacy, this will behave as a confounding factor in broad analyses. Those treated with immunosuppressant medication already exhibit one of the best correlations to efficacy. Ultimately then, the question in these cases should not be if individuals who experience irAEs and have steroids fair better than individuals who do not have steroids, but rather, will the introduction of steroids worsen an individual’s clinical course of tumor control? It is notable that some treatment combinations with chemotherapy and ICIs use corticosteroids as premedication within the treatment protocol where therapeutic benefit is seen, but the question remains as to whether it could be enhanced if steroids were to be avoided. Murine studies (MC38 xenograft, anti-PD-1, and anti-CTLA-4-treated mice) have indicated prednisolone does significantly diminish the antitumor effect of anti-PD-1 and anti-CTLA-4-treated mice.^43^ Studies carried out in patients with advanced non-small-cell lung carcinomas (NSCLC), melanoma, or urothelial carcinoma indicated that it is likely that the administration of corticosteroids decreases the efficacy of ICIs as oncological treatments.^44,45^ Studies by Svaton et al. indicated that there is a significant increase in disease progression in advanced NSCLC patients administered corticosteroids at the time of nivolumab treatment; additionally, they concluded that the administration of nonsteroidal anti-inflammatory drugs (NSAIDs) may improve outcomes for ICI patients.^46^

### Anticytokine Agents

Infliximab is an anti-TNFα agent, which is commonly prescribed to treat autoimmune diseases such as psoriasis and Crohn’s disease; it is now commonly prescribed to treat irAEs following ICI therapy when patients are nonresponsive to corticosteroid treatment.^34^*In vivo* animal studies indicate that infliximab administration in combination with ICIs increased the tumor response of ICIs and improved symptoms of colitis.^47,48^ Additionally, it was reported that gastrointestinal inflammation was successfully treated without recurrence in five patients with different primary malignancies who were administered mono or combinational ICI therapy and also administered infliximab, subsequently allowing their further ICI therapy to be tolerated.^42^ Due to the fact that the mechanism of irAEs is similar irrespective of tissue type, anti-TNFα therapies have been shown to be effective in patients with multiple different types of steroid refractory irAEs and are currently in trials outside of the gastrointestinal setting, e.g., pneumonitis. Dimitriou et al. retrospectively concluded that immunomodulatory drugs such as anti-TNFα and anti-IL-6 agents had no effect on the efficacy of ICIs in melanoma patients.^49^ There have been some cases of hepatitis reported with infliximab, and it is therefore often avoided in cases of hepatitis, though evolving evidence is suggestive of benefit in certain cases, particularly in the treatment of refractory hepatitis and sclerosing cholangitis. An individual case indicated that a prostate adenocarcinoma patient administered infliximab to treat ICI-induced colitis then subsequently developed hepatoxicity believed to be due to the administration of infliximab.^50^ However, Araujo et al. retrospectively investigated 56 ICI patients with various malignancies who were also administered infliximab and observed that there is no indication that the concurrent administration of infliximab with ICIs increases ALT, AST, and total bilirubin levels.^51^ Infliximab should not necessarily be ruled out for ICI patients with hepatotoxicity and instead used with caution as it may in fact improve patient overall clinical outcomes by enabling them to sustain their ICI therapy by improving/preventing other irAEs such as colitis, which may have led to the discontinuation of their therapy.^52^ Further investigation into whether the risk of infliximab-induced liver injury outweighs the potential improved overall clinical outcomes for ICI patients is needed. Vedolizumab is a monoclonal antibody therapy that targets α4β7 in the gut, and there is strong evidence that it is effective in the treatment of ICI-induced enterocolitis. The impact on a patient’s response to their ICI therapy is not well-defined, but given the gut specific nature of its mechanism of action, it is less likely to cause a decrease in efficacy in the majority of malignancies.^53,54^ α4β7 expressing colorectal cancers and/or metastasis may represent a distinct subset of cancers in this respect and may need to be carefully considered. The role of IL-17 from Th17 cells in terms of protumorigenic or antitumor effects in the tumor microenvironment (TME) is complex and most likely context dependent.^55^ Liu et al. used murine models to propose that anti-IL-17 agents may enhance the tumor responses to PD-1 inhibitor therapy in microsatellite stable colorectal cancer.^56^ In murine models, it has been proposed that anti-IL-17A antibodies reduce thyroid irAEs in ICI-treated mice without negatively affecting the antitumor efficacy of the ICI.^57^ Additionally, a number of cases have now been reported where the IL-17A blocking agents secukinumab and ixekuzumab have successfully treated psoriasis-like dermatologic toxicity after pembrolizumab and atezolizumab therapy.^58−61^ In noncancer patients treated with secukinumab for diseases such as psoriasis, psoriatic arthritis, and ankylosing spondylitis, the incidence of secukinumab-related colitis and irritable bowel disease has been investigated. In a meta-analysis, new onset of colitis or irritable bowel disease cases occurred in less than 1% of patients; however, exacerbation of disease was found to occur at a much higher rate with 11/48 patients with pre-existing irritable bowel disease.^62^ A similar rate (approximately 0.5%) was observed in a 21 trial meta-analysis of ixekuzumab-treated individuals.^63^ There is evidence that IL-17A has an important mechanistic role in the protection and maintenance of epithelial barriers in the intestinal mucosa; therefore, there is concern as to whether the risk of already common gastrointestinal irAEs may be exacerbated after administration of anti-IL-17A therapies.^64^

### Sulfasalazine

As part of the irAE profile, ICI therapy can induce arthritis as a *de novo* irAE and lead to exacerbation of existing inflammatory arthritides.^65^ As with most irAEs, the clinical algorithm by which ICI-associated arthritis is treated is effectively lifted from the parallel noniatrogenic autoimmune disease. Indeed, ICI-associated arthritis is commonly successfully treated with corticosteroids, NSAIDs, or disease-modifying antirheumatic drugs.^65^ The prodrug sulfasalazine is classed as a disease-modifying antirheumatic drug and is metabolized by colonic bacteria to its constituents the sulfonamide antibiotic sulfapyridine and the anti-inflammatory mesalazine. In 2018, Ford et al. presented a case series detailing outcomes of four metastatic melanoma patients receiving sulfasalazine for amelioration of ICI-induced (anti-PD-1 ± prior anti-CTLA-4) arthritis. These patients then presented with delayed adverse effects such as fever, maculopapular rash, nausea, diarrhea, abnormal liver function tests, and elevated CRP. Resolution of these ill effects was seen upon discontinuation of sulfasalazine, indicating sulfasalazine was the causative agent for these secondary irAEs and that they were not the direct effects of ICIs *per se*. From subsequent *ex vivo* mechanistic workup of these metastatic melanoma patients, it was determined that all three patients exhibited positive lymphocyte responses against active ingredients or downstream derivatives of sulfasalazine within *in vitro* diagnostic assays outlined in Hammond et al. indicating the presence of an established type IV hypersensitivity reaction where drug responsive CD4+ T-cells were generated.^23,66^ Given the occurrence of hypersensitivity in sequential patients treated in this cohort, there is a suggestion that the presence of ICI reduces hypersensitivity tolerance when given concomitantly with drugs known to have a hypersensitive propensity.

## Pre-Existing/Incidental Medications and Chemicals

Due to the comorbidity burden of many of the cancer subtypes treated with ICIs, a background of polypharmacy is common in patient populations subject to IO therapy. Numerous medications have been surveyed for associations with altered pharmacodynamic profiles in ICI treatment.^67^ Key classes of compounds that are administered with prevalence in both general and specific oncology populations are systemic NSAIDs, antibiotics, and gastric acid suppressants. Proton pump inhibitors (PPIs) are a key component of the latter class, which irreversibly inhibit stomach H^+^/K^+^ ATPase proton pumps and are widely prescribed to treat gastric ulcers, gastroesophageal reflux disease, and acid reflux. They are also commonly used to provide “gastric protection” in patients treated with corticosteroids, a drug class commonly used to treat irAEs. Associations have been made between systemic antibiotics and PPIs and poor prognoses of patients, with significantly worse outcomes, particularly in terms of overall survival but also progression free survival and objective remission rate.^67−70^ The reason for this has not been mechanistically delineated to date; however, it is suspected to be related to microbiota modifying the qualities of these drugs. Indeed, the composition and status of the gut microbiome and effects of ICIs have a complex but intimate relationship. This was competently demonstrated in tumor bearing mice for anti-CTLA-4, anti-PD-1, and anti-PD-L1^71−73^ with the transfer of favorable faecal microbiome proving efficacious in all cases. Moreover, antibiotic coadministration was detrimental to efficacy in several of these models.^71,73^ The human picture is more complex, but there is great interest in this area in terms of therapeutic exploitation and tolerability nevertheless.^40,74^ Considerations in this area include the qualitative changes imposed by medications, for example, broad or narrow spectrum of antibiotics, as well as duration and timing of administration. Reports of generally well-tolerated drugs eliciting adverse reactions of immune etiology within patients following ICI therapy are notably accruing and may offer explanation for a subset of organ specific irAEs.

### PPIs

In the wider population, PPIs are known to be associated with kidney injury, where in a study of 10 000 patients it was concluded that the administration of PPIs was independently associated with a 20–50% higher risk of incident chronic kidney disease.^75^ Acute kidney injury occurs in 2–5% of ICI-treated patients, often presenting as tubulointerstitial nephritis.^76−78^ In 2018, a case series outlined six lung cancer patients previously treated with (and tolerant to) omeprazole, lansoprazole, and NSAIDs (ibuprofen) who subsequently experienced acute interstitial nephritis (AIN) following commencement of anti-PD-1 therapy.^79,80^ Additional case studies on PPIs behaving in such a fashion have emerged since; one particular case outlined the breakdown of tolerance to lansoprazole after nivolumab administration, which had been safely administered to a lung cancer patient for four years prior, subsequently resulting in kidney injury.^79^ Manohar et al. reported that 11/14 patients with either melanoma, breast cancer, lung adenocarcinoma, or chronic lymphocytic leukemia treated with ICIs suffered ICI-AIN but were also administered PPIs.^81^ Out of the 11 patients, 8 ceased PPI use, and 5/8 of these patients had kidney function returning back to normal when PPIs were discontinued.^81^ This has since become an area that has received attention, not least due to the frequency of PPI administration in IO populations. Indeed, multiple retrospective studies evaluating large patient cohorts have now identified PPI administration as a significant risk factor for acute kidney injury in ICI-treated patients for a range of malignancies.^82−84^ Notably, Gupta et al. retrospectively investigated ICI-associated acute kidney injury in 429 patients who received ICI treatment and developed acute kidney injury and compared them to 429 patients who received ICI therapy without kidney injury; from each patient group, malignancies varied including melanoma, lung, and genitourinary.^83^ It was concluded that the administration of PPIs was associated with an increased risk of acute kidney injury in ICI therapy patients with 208/429 ICI-acute kidney injury patients receiving PPIs at the time of injury.^83^ In many of these cases, PPIs were previously tolerated, and the irAE in the form of AIN was observed once immunotherapy was initiated. It is conceivable that this is due to the threshold for these concomitant medications to cause AIN being lowered after the administration of ICIs via either the activation and mobilization of a drug-specific memory T-cell compartment or alternatively due to a lowered threshold for elicitation of extensive *de novo* T-cell responses.^83,85^ In addition to kidney injury, there have also been reports of skin toxicities in the form of SJS events linked to the administration of PPIs concomitantly during ICI therapy. Lin et al. reported a stage IV lung adenocarcinoma patient diagnosed with SJS after nivolumab and esomeprazole administration; esomeprazole was confirmed to be the culprit medication for the reaction as after rechallenge with esomeprazole 3 months post the initial SJS event, SJS recurred.^86^

### Antibiotics, NSAIDs, Paracetamol

Other concomitant medications which historically cause kidney injury such as NSAIDs and antibiotics were also investigated in the Gupta et al. study; however, they reported no significant increases were found in ICI-treated patients with kidney injury also treated with these medications.^83^ Martínez Valenzuela et al. reported two cases of acute tubulointerstitial nephritis. The first patient diagnosed with NSCLC was treated with carboplatin, paclitaxel, nivolumab, and NSAIDs; 7 days after NSAID initiation they were admitted with high-grade fever, and subsequent diagnostic testing concluded acute tubulointerstitial nephritis.^87^ The second patient was treated with nivolumab for stage IV clear cell renal carcinoma with lung and liver metastases; they were admitted due to acute kidney injury 5 days after they concurrently took ibuprofen.^87^ Kawada et al. presented a case where TEN occurred in a stage IV NSCLC patient who was administered pembrolizumab alongside sulbactam/ampicillin, ceftriaxone, penicillin, metronidazole, and paracetamol.^88^ Positive LTTs were observed for pembrolizumab, paracetamol, and metronidazole only; however, after rechallenge with pembrolizumab due to cancer progression, no subsequent adverse cutaneous reactions occurred. It was therefore inferred that the causative agents may have been metronidazole and/or paracetamol. In 2020, Watanabe et al. released a case report of an advanced oral melanoma patient who was administered nivolumab one month prior to suffering TEN; the causative agent of the reaction was deemed as paracetamol as positive LTTs to this drug were observed.^104^ Lomax et al. documented a melanoma patient who had a confirmed case of early TEN after receiving cephalexin 12 days and throughout pembrolizumab treatment.^93^ The patient was successfully rechallenged with pembrolizumab without a repeated skin reaction, indicating an immunological drug–drug interaction between the coadministered cephalexin and pembrolizumab led to the TEN-like reaction in this patient.^93^ Additionally, reports of lung cancer patients suffering from hypersensitivity reactions caused by trimethoprim/sulfamethoxazole after ICI administration have been reported.^107,108^ Kimura et al. reported a lung cancer patient suffering from interstitial pneumonitis caused by a combination of anti-CTLA-4 and PD-1 therapy.^107^ This adverse reaction was subsequently treated with trimethoprim/sulfamethoxazole in combination with prednisolone; however, this caused a drug-induced hypersensitivity reaction where the stimulation index (SI) was 13.6 for trimethoprim/sulfamethoxazole in an LTT.^107^ Additionally, Urasaki et al. reported a metastatic kidney cancer patient suffered grade 3 interstitial pneumonitis after anti-CTLA-4 and PD-1 therapy, who was subsequently also treated with trimethoprim/sulfamethoxazole in combination with prednisolone; this then induced hypotensive shock accompanied with cytokine release and drug-induced hypersensitivity syndrome.^108^

### Amidotrizoate (Iodinated Contrast Media)

A case study outlined the observation of an immunologically driven adverse interaction between atezolizumab with the iodinated contrast media amidotrizoate in a patient treated for metastatic renal cell carcinoma.^22^ The introduction of atezolizumab in this clinical case study appeared to shift the immunological perception of amidotrizoate from tolerance/ignorance to a state of elicitation, resulting in a severe, idiosyncratic, and cutaneous reaction. The immediacy of the reaction (occurring within hours of exposure) does not correspond with the time required for the initiation of a *de novo* priming response of T-cells; therefore, a logical deduction is that the initial reaction represents the ICI-mediated transition to activation of a senescent memory component accrued through repeated historical exposure to amidotrizoate.

### Tattoos

There are several cases where IO patients with tattoos have experienced cutaneous reactions following the initiation of ICI therapy.^116−118^ In one particularly striking case, a patient treated with durvalumab for the treatment of adrenal and cerebral metastatic lung cancer experienced sarcoidosis only on the black ink parts of their tattoos, which was present prior to ICI therapy without issue for over 40 years.^118^ Tattoos are notoriously inconsistent mixtures of chemicals, so it is unlikely that the causative chemical will be delineated. In these cases, the reactions observed in the patients ceased after the discontinuation of ICI therapy; therefore, this demonstrates the reversibility of these specific reactions in patients where antigen presence is maintained.

## Combinational Oncological Therapy

In recent years, combinational cancer therapy has been administered to improve clinical outcomes for patients. Combinational therapy may involve the administration of multiple anticancer agents including combinations of ICIs or ICIs administered in combination with other anticancer agents such as TKIs.^119^ Indeed, the combination of ICI therapy and other oncological agents could benefit cancer patients in terms of their overall survival; however, the unknown drug–drug interactions with combinational therapy may lead to an increased risk of irAEs. The risk-benefit ratio for patients will certainly be important for future decision making in the administration of combination oncological therapies.

### EGFR Inhibitors and VEGF Inhibitors

TKIs inhibit cancer cell proliferation by their competition with adenosine triphosphate (ATP) for the ATP binding site of protein tyrosine kinase and subsequent reduction of tyrosine kinase phosphorylation.^120^ Clinical trial results indicate that TKIs and ICIs have a synergistic antitumor effect with improvements in progression-free survival in sarcoma patients treated with nivolumab and sunitinib and improvements in progression free survival and overall survival in renal cell carcinoma patients treated with nivolumab and cabozantinib.^11,121^ Additionally, there is an increasing body of evidence suggesting that antiangiogenic drugs such as anlotinib used in combination with ICIs offer antitumor activity for patients with NSCLC.^122^

Osimertinib is a third-generation epidermal growth factor receptor (EGFR) TKI, which was first approved for the treatment of EGFR T790M mutation positive NSCLC. Osimertinib forms an irreversible covalent bond at the cysteine-797 residue in the ATP binding site of mutant EGFR (Leonetti, Sharma, Minari, Perego, Giovannetti, and Tiseo, 2019).^300^ After the sequential dosing of osimertinib after ICI therapy, specifically anti-PD-1 and anti-PD-L1 monoclonal antibody therapies, a range of irAEs have been reported. These adverse side effects include interstitial lung disease and hepatoxicity, which have been observed at high levels in patients who received osimertinib immediately after nivolumab therapy.^97,98,100^ Oshima et al. retrospectively concluded that the combination of the PD-1 inhibitor nivolumab and EGFR TKIs in NSCLC patients significantly increased the risk of interstitial pneumonitis.^123^ Gianni et al. reported an NSCLC patient who suffered from grade 3 hepatoxicity after treatment with chemotherapy and pembrolizumab followed by the administered osimertinib 10 days later; after recovery, the patient developed a subsequent grade 3 hepatoxic reaction alongside SJS when osimertinib was restarted.^101^ In 2020, a phase Ib trial was reported on where the combination of osimertinib with other agents such as selumetinib, savolitinib, and durvalumab was assessed in patients with EGFR mutant NSCLC and disease progression with previous EGFR-TKI administration.^103^ This study concluded that osimertinib in combination with selumetinib or savolitinib was tolerable; however, when given in combination with durvalumab, this led to interstitial lung disease in 22% (5/23) of patients.^103^ Notably, a case was reported where an NSCLC patient received chemotherapy in combination with pembrolizumab where no irAEs were observed; however when osimertinib was administered 3 weeks later, the patient developed a range of irAEs including fatal TEN.^99^ An additional study stated that 24% of EGFR mutant NSCLC patients who received osimertinib within 3 months after anti-PD-(L)1 therapy suffered from severe irAEs.^124^ There were no severe irAEs reported in EGFR mutant NSCLC patients who were administered osimertinib followed by PD-(L)1 therapy or received other EGFR TKIs such as afatinib or erlotinib after PD(L)1 therapy.^124^

### Dacarbazine

Other oncological agents which are used in combination with ICIs include the alkylating agent dacarbazine, which has been shown to increase overall survival in previously untreated metastatic melanoma patients when treated with the CTLA-4 inhibitor ipilimumab and dacarbazine when compared to patients treated with dacarbazine alone.^125^ However, this is accompanied by a high occurrence of dacarbazine-induced liver injury, Robert et al. reported that 56.4% of metastatic melanoma patients administered dacarbazine and ipilimumab suffered from grade 3 or 4 adverse events compared to 27.5% of patients treated with dacarbazine and a placebo.^125^ A phase II study where previously untreated, unresectable, or metastatic melanoma patients were administered ipilimumab plus dacarbazine found that this combination was intolerable due to high-grade liver toxicities.^96^ Yamazaki et al. reported that 93% of patients in the trial had irAEs predominantly liver (80%) and skin (67%) toxicities.^96^

### Abemaciclib

Abemaciclib is a CDk4/6 inhibitor that has been investigated clinically in combination with ICI therapy, specifically anti-PD-1 therapy. A phase Ib trial where KRAS mutant or squamous NSCLC patients received abemaciclib in combination with pembrolizumab concluded that the combination had remarkable antitumor activity; however, this was comparable to pembrolizumab monotherapy. Additionally, the combination resulted in a higher rate of transaminase elevations and pneumonitis.^90^ Additionally, in a phase II trial of nivolumab in combination with abemaciclib plus endocrine therapy in patients with hormone receptor-positive, human epidermal growth factor receptor-2 negative metastatic breast cancer resulted in severe and prolonged irAEs.^89^ This study was terminated early due to safety concerns; 10/17 patients developed grade ≥3 liver-related adverse events. Additionally, one treatment-related death from interstitial lung disease occurred.^89^ Masuda et al. concluded that their findings suggested that the suppression of Treg proliferation and production of proinflammatory cytokines (TNF and IL-11) as a result of the addition of abemaciclib to nivolumab therapy were the cause of the irAEs.^89^

### Sotorasib

Sotorasib is a covalently binding KRAS inhibitor that has been assessed for the treatment of NSCLC in conjunction with ICIs. Begum et al. reported a case where a NSCLC patient suffered severe hepatotoxicity after the administration of sotorasib after prior treatment with carboplatin–pemetrexed–pembrolizumab.^106^ Two significantly striking retrospective case studies assessed the safety of sequential ICI and sotorasib therapy. Rakshit et al. determined that in NSCLC patients treated with sotorasib with 28/32 patients receiving ICI therapy prior, of the 28 patients grade 3 hepatoxicity was observed in 3/4 who received ICIs within 30 days, 7/11 who received ICIs within 31–90 days, and 0/13 in patients who received ICIs >90 days.^126^ Risk of hepatotoxicity was higher in patients who received sotorasib within 90 days of ICI treatment, whereas none of the four patients without prior ICI exposure developed any hepatotoxicity. Chour et al. also retrospectively investigated sotorasib administration after anti-PD-(L)1 treatment in NSCLC patients and concluded that severe sotorasib-related adverse events including hepatotoxicity were significantly more frequent in the patients who received sequential anti-PD-(L)1 and sotorasib therapy compared to patients who did not (control group), with severe sotorasib-related hepatotoxicity being 3 times more frequent in the sequence group compared with that in the control group (33 versus 11%, *p* = 0.006).^127^ These reports highlight the importance and implications for the sequencing and timing of these oncological agents. In addition to raising the question of importance of wash out periods, is it both the presence of ICIs and also the timing from the last administration that have an impact on the tolerability of sotorasib.

### BRAF Inhibitors

Due to ICIs and the BRAF inhibitor vemurafenib improving overall survival for patients with advanced melanoma independently, the benefit of the combination of the two agents has been trialled also due to speculation of BRAF inhibitors potentially enhancing antigen presentation and immune cell function.^128^ In 2013, it was first reported that patients with metastatic melanoma with a BRAF V600 mutation in a phase I trial treated with ipilimumab and vemurafenib suffered from hepatoxicity where aminotransferase levels elevated to a grade 3 toxicity in the majority of patients; additionally, a study conducted in 2018 evaluated vemurafenib-treated Japanese patients with metastatic melanoma; 6/7 patients who suffered severe skin reactions received PD-1 inhibitor therapy before vemurafenib.^109,115^ Dabrafenib is also a BRAF inhibitor; in phase I/II trials, BRAF V600E/K-mutated melanoma patients received dabrafenib and ipilimumab double therapy or dabrafenib, trametinib, and ipilimumab triple therapy.^95^ In 2/7 patients receiving the triple therapy, severe colon toxicity was observed.^95^

## Antidrug Antibodies

An intriguing and perhaps less obvious example of ICIs altering the immunological perception of therapeutics is through the modulation of antidrug antibody (ADA) formation. An excellent example of this is the combination of anti-CTLA-4 and anti-PD-1 inhibitors; when these agents have been used in combination, perturbation of pharmacokinetic parameters has been reported, with a 24% increase in clearance of nivolumab observed in combinatorial use with ipilimumab relative to monotherapy.^129^ Initially, this was not considered clinically relevant due to the lack of detection of a detriment to efficacy. However, later speculation that the enhanced clearance was attributable to the increase in antidrug antibodies^130^ indicates yet another immunologically driven drug–drug interaction, this time with pharmacokinetic ramifications. Theoretical effects on pharmacodynamics are obvious; if clearance is adequately increased and/or neutralizing antibodies are formed, then this will be to the detriment of the pharmacokinetic profile and thus efficacy of the therapeutic. However, ADAs have not been studied in enough depth within the IO field to say with vindication if this theoretical concern is relevant in the clinic. Indeed, practical clinical experience even with monotherapy does not yield clear signals, in part due to a lack of comprehensive studies.^131,132^ There are, however, a small number of reports that correlate ADA formation to ICIs in monotherapy to poorer overall survival outcomes with ipilimumab^133^ and atezolizumab. Finally, an important caveat and major impediment in determining the effects of ADAs on efficacy lies in the comparison of ADA positive vs non-ADA positive individuals in terms of efficacy; the promotion of ADAs in the first place could be interpreted as pharmacodynamic activity in itself and so could be a surrogate of efficacy to some degree. The true impact of ICI-induced ADA formation may therefore not be possible to assess appropriately against another agent that has immune-modulatory or even tumor function but may come to light when a biological therapeutic with a distinct mechanism of action is investigated.

Even less well-characterized is how a possible induction of ADAs might lead to alterations of the toxicological profile. It is well-documented that circulating immunoglobulins can contribute to hypersensitivity reactions through various mechanisms,^134^ with the classic immediate/type I causing anaphylaxis often a key concern with biologicals. The true effect of immunomodulatory qualities on this aspect of immunogenicity is yet to be delineated. However, logically, the immunomodulatory qualities of ICIs might promote such issues with concomitant biologicals.

## Approaches to Address the Issue (Timing, Drug Choice, Patient Stratification)

**Figure 4:**
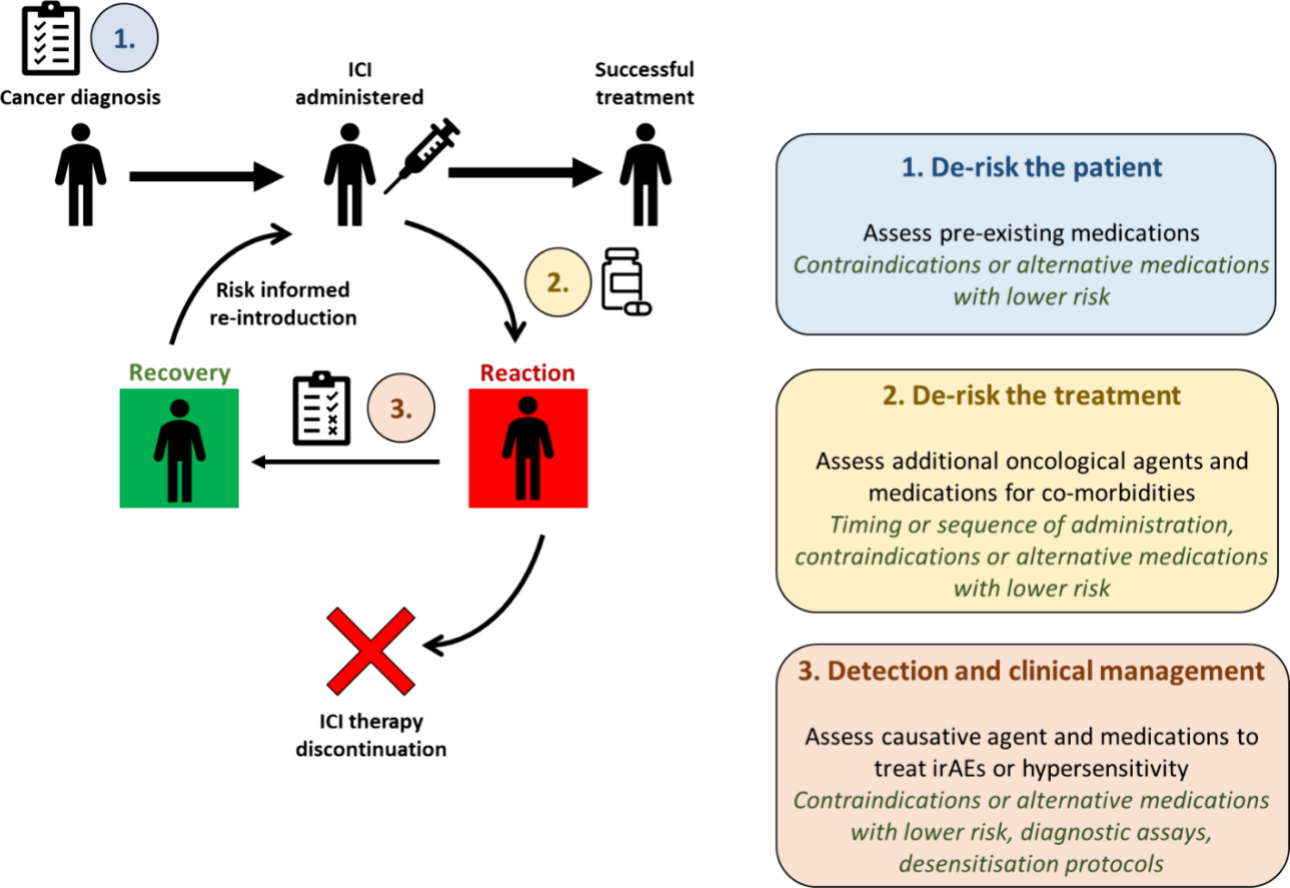
Hypothetical immune-oncology patient treated with immune checkpoint inhibitor (ICI) therapy following pretreatment screening, administration of ICI therapy, immune-related adverse event (irAE) or hypersensitivity reaction, and either ICI therapy discontinuation or recovery and readministration of ICI therapy. To mitigate the risks of irAEs and hypersensitivity reactions caused by concomitantly administered drugs during ICI therapy, there are potentially three areas that can be addressed. First, derisking the patient by assessing their pre-existing medications. Second, derisking the treatment by assessing additional oncological agents and other medications likely to be introduced during treatment. Lastly, detecting and clinically managing the causative agent of the irAE/hypersensitivity reaction and assessing medications administered for treatment of such reactions.

Given the emerging body of evidence for ICI-induced drug hypersensitivity reactions for drugs as a form of irAE, it is clear that these types of reactions are a serious problem for IO patients. These reactions may prevent patients from continuing their successful cancer treatment due to discontinuation of therapy, which could be catastrophic for patients with no alternative treatment options. Due consideration must be given to whether this component of the safety profile could be improved. Several time points in a patient’s journey do appear tractable with regards to this (Figure 4) as outlined and discussed in detail below.

### Detection and Management

Due to their idiosyncratic nature, an undesirable truth of drug hypersensitivity reactions is that the beginning of the journey to the understanding of how, when, and who they occur in begins with the frontline clinical detection of toxicity signals, often in late phase or postmarketing surveillance. The suspicion of what is effectively an immunological drug–drug interaction underlying a hypersensitivity reaction presents a nuanced version of this same challenge. Indexed above (Table 2) are numerous examples where the coadministration of ICI with a second medication results in an intolerable adverse outcome. The examples outlined represent some of the best defined cases of ICI-induced drug hypersensitivity to date, with the etiology of the reaction supported by investigative work up or extensive clinical evidence. It is noteworthy that several trials have been pivotal in determining the tolerability of combination therapies in certain settings^10,96,135^ In real-world practice on a background of heterogeneous polypharmacy in patient populations, it is probable that this mode of toxicity is under-reported and somewhat underappreciated at present. Part of the reason for this is the challenging nature of irAEs in terms of prevalence and presentation. Indeed, several high-profile instances of severe reactions outlined in (Table 2) were first attributed to the direct irAE profile of the ICI itself, which following the readministration of the true antigen, led to the patient experiencing a second bout of severe adverse reaction before the true nature of the reaction was recognized.^22,86^ The initial steps toward mitigating these reactions is therefore through diligent pharmacovigilance; identifying reactions when they occur in the clinic and continual evaluation of combinations of drugs, retrospective evaluation of patient cohorts may also help to identify signals with compounds that have been missed, especially as studies become large enough to adequately power such observations. In the first instance, reviewing data on outcomes of patients treated with historically relevant drugs with known liabilities for hypersensitivity in indications might be productive in this regard. With respect to new onset reactions, diagnostic procedures for hypersensitivity tend to rely extensively on a robust clinical characterization and work up, which can include *in vivo* evaluation such as skin patch tests, rechallenge etc. where appropriate. Further to this, as discussed in Hammond et al., various investigational tools (LTTs, ELISpot etc.) are available for *in vitro* assessment of reactions and have been employed effectively to aid determination of causality in a safe and accurate manner in several reports to date.^136,22,23^ Where the ICI is not the culpable agent, this may aid the successful reintroduction of the ICI in the absence of the offending therapeutic.

**Table 2:** Cases of Immune-Related Adverse Events Caused by Concomitant Medications after Immune Checkpoint Inhibitor Therapy^a^

**Small molecular weight drug administered**	**Paper**	**Reference**	**Immune checkpoint inhibitors administered**	**Immune-related adverse event**
Abemaciclib	Clinical trial report	Masuda, Tsurutani [^89^]	Nivolumab	Fatal interstitial lung disease
		Pujol, Vansteenkiste [^90^]	Pembrolizumab	Pneumonitis
Allopurinol	Case report	Griffin, Brooke [^91^]	Nivolumab	TEN
Amidotrizoate	Case report/experimental study	Hammond, Olsson-Brown [^22^]	Atezolizumab	SJS
Capmatinib	Case report	Sisi, Vitale [^92^]	Pembrolizumab	Drug-induced liver injury
Cephalexin	Case report	Lomax, McQuillan [^93^]	Pembrolizumab	Acute TEN
Crizotinib	Clinical trial report	Lin, Chin [^94^]	Pembrolizumab, nivolumab, ipilimumab, atezolizumab	Hepatotoxicity
Dabrafenib	Clinical trial report	Minor, Puzanov [^95^]	Ipilimumab	Gastrointestinal toxicity
Dacarbazine	Case report	Yamazaki, Uhara [^96^]	Ipilimumab	Drug-induced liver injury
Esomeprazole	Case report	Lin, Yang [^86^]	Nivolumab	SJS
Ibuprofen	Case report	Shirali, Perazella [^80^]	Pembrolizumab, Nivolumab	Acute tubulointerstitial nephritis
	Two case reports	Martínez Valenzuela, Antón [^87^]	Nivolumab	Acute tubulointerstitial nephritis
Omeprazole	Case report	Shirali, Perazella [^80^]	Pembrolizumab, Nivolumab	Acute tubulointerstitial nephritis
Osimertinib	Case report	Kotake, Murakami [^97^]	Nivolumab	Interstitial lung disease
		Takakuwa, Oguri [^98^]	Nivolumab	Interstitial lung disease
		Cui, Cotter [^99^]	Pembrolizumab	Fatal TEN
		Yamaguchi, Kaira [^100^]	Nivolumab	Hepatotoxicity
		Gianni, Bronte [^101^]	Pembrolizumab	Hepatoxicity and SJS
		Lopez, Hagopian [^102^]	Pembrolizumab	SJS/TEN
	Clinical trial report	Oxnard, Yang [^103^]	Nivolumab	Interstitial lung disease
Lansoprazole	Case report	Koda, Watanabe [^79^]	Nivolumab	Acute tubulointerstitial nephritis
Metronidazole	Case report	Kawada, Nobeyama [^88^]	Pembrolizumab	TEN
Paracetamol	Case report	Watanabe, Yamaguchi [^104^]	Nivolumab	TEN
	Case report	Kawada, Nobeyama [^88^]	Pembrolizumab	TEN
Selpercatinib	Clinical trial report	McCoach, Rolfo [^105^]	Atezolizumab, avelumab, cemiplimab, durvalumab, nivolumab, pembrolizumab, and spartalizumab	Maculopapular rash, thrombocytopenia, increased AST or ALT
Sotorasib	Case report	Begum, Goldin [^106^]	Pembrolizumab	Hepatotoxicity
Sulfasalazine	Case report	Ford, Sahbudin [^66^]	Pembrolizumab and ipilimumab	Cutaneous reactions
	Experimental study	Hammond, Olsson-Brown [^23^]		
Trimethoprim/sulfamethoxazole	Case report	Kimura, Hasegawa [^107^]	Pembrolizumab	SJS
	Case report	Urasaki, Ono [^108^]	Nivolumab, ipilimumab	Drug-induced hypersensitivity syndrome
Vemurafenib	Clinical trial report	Ribas, Hodi [^109^]	Ipilimumab	Hepatotoxicity
	Case report	Johnson, Wallender [^110^]	Nivolumab and pembrolizumab	Severe cutaneous reactions
	Case report	Imafuku, Yoshino [^111^]	Nivolumab	Severe cutaneous reaction
	Case report	Tsuboi, Yoshino [^112^]	Nivolumab	Severe cutaneous reactions
	Case report	Urosevic-Maiwald, Mangana [^113^]	Ipilimumab and pembrolizumab	Systemic inflammatory reaction syndrome
	Case report	Lamiaux, Scalbert [^114^]	Pembrolizumab, ipilimumab and nivolumab	One case of SJS and four cases of severe DRESS
	Case report	Uhara, Kiyohara [^115^]	Nivolumab and pembrolizumab	Cutaneous reactions

a SJS: Stevens-Johnson syndrome, TEN: toxic epidermal necrolysis, DRESS: drug reaction with eosinophilia and systemic symptoms syndrome, ALT: alanine aminotransferase, AST: aspartate aminotransferase.

### Derisking the Patient

Armed with knowledge acquired from pharmacovigilance/clinical experience, as patterns of risk with particular comedications begin to emerge, it may be possible to effectively triage patients prior to treatment to identify potentially problematic medications in terms of safety/efficacy. With sufficient evidence to build a risk-benefit profile, it may be possible to contraindicate some therapeutics at this stage or refer patients to alternative concomitant medications that have precedence of a lower risk profile in terms of inducing drug hypersensitivity reactions. Where such courses of action are not possible/necessary, identification of potential hazards at this stage may help inform patient and physicians alike and may hasten/direct suspicion and decision making should a reaction be observed later down the line. Whether derisking patients in this manner will be effective remains to be addressed. Association of specific HLA alleles with increased likelihood of drug hypersensitivity reactions is reported for drugs such as abacavir (HLA-B*57:01) and carbamazepine (HLA-B*15:02). Currently, it is unknown as to whether the coadministration of these drugs with ICIs in patients who possess these HLA-risk alleles will enhance the likelihood of irAEs to these coadministered drugs. However, this is also an important concept that must be considered and investigated further.

### Derisking the Treatment

The subsequent step in managing these toxicities is to derisk the treatment itself by carefully considering additional agents likely to be introduced during and in succession to ICI treatment. The liabilities of monotherapy with the secondary agent should be considered, especially if the toxicity profile might induce lesions that could be exacerbated by the mechanism of action of ICIs. Optimization of treatment algorithms for various commonly used medications should be pursued here, reducing the risk of introducing problematic agents during the course of treatment in terms of toxicity and efficacy profile.

One of the most important aspects of this is combinatorial or sequential oncology treatment. Where the adjunctive/additional combinatorial agent is established, there is a benefit of understanding the baseline toxicity profile, and it is sometimes possible to envisage potential synergistic toxicity, e.g., GI toxicity with chemotherapy, for example, with paclitaxel plus cisplatin,^137,138^ and skin toxicity with TKIs, for example, afatinib, erlotinib, and gefitinib,^139−142^ may be exacerbated. However, a different challenge manifests for a novel therapeutic in the early stages of clinical development. This is particularly important when drugs are primarily intended to be used alongside immunotherapy. Good laboratory practice toxicity studies are not generally performed for combinations where one agent is clinically established, and immune reactions of this type are often of limited translational value in any case. In both scenarios, it is desirable to optimize the safety profile as efficiently as possible. These combinatorial approaches are often evaluated within well-monitored clinical trials and have led to the identification of adjunctive therapies that are not tolerated for example ipilimumab plus dacarbazine in melanoma patients.^96^

Where the combination is part of an intentional therapeutic regimen, but rather incidental, the solution can extend to revised treatment algorithms for agents to be introduced. A prime example of this is sulfasalazine, which is not commonly administered for ICI-induced rheumatoid events due to clinical and experimental evidence that indicated the hypersensitivity issue.^23,66^

Another key aspect with regard to drugs introduced during treatment is the temporal relationship of any ICI-imposed effects. A particularly pertinent question is what the effective washout period of ICI modulation is at what time does the risk of hypersensitivity with the introduction of an additional agent return to approximately baseline for a given patient? Certainly, the sequence of ICI administration appears to affect the tolerability of given combinations; osimertinib before ICI therapy is deemed safer than when given in combination or after immunotherapy, which has evidence of causing severe and fatal irAEs.^97−100,103^ The long half-life of antibodies (3–4 weeks)^143,144^ raises the possibility that the effect of IO agents on the immunological perception of compounds for a given patient may extend long past the final administration, and therefore, introduction of new agents following administration of ICIs is likely to be less favorable for some time. As described by Watanabe et al., the effects of ICI therapy have the potential to lead to long-lasting lymphocyte activation and gradual and sustained suppression of Tregs, which can subsequently lead to hypersensitivity reactions to concomitant medications weeks after ICI therapy discontinuation.^104^ Additionally, this is consistent with reports of various pharmaceuticals exhibiting poor tolerability when administered in sequence with ICIs.^66,115^

A further long-term goal might even be the refinement of the IO therapies. At present, the selection of blocking monoclonal antibodies on the market for IO therapy represents the first generation of the immune checkpoint blockade era. These antibodies systemically block the target, and so, their extratumoral effects on immune regulation are widespread, extensive, and of relevance in the context of adverse effects. Multiple waves of newer therapeutic approaches are in development now, with the total number of prospective IO therapeutics growing exponentially; by 2020, both PD-1 and PD-L1 were the intended therapeutic targets of over 100 distinct IO agents at various stages of development. One of the key themes with the coming iterations may indeed be to increase the efficacy or therapeutic index of such agents with respect to on/off-tumor activity. Approaches to this pursued to date include modifications to conventional antibody constructs (multivalency, prodrug-like behavior, e.g., pacmilimab), and alternative platforms deliver the intended disruption of the antibody (e.g., oligonucleotides selectively delivered to tumor cells by advanced modality platforms or alternative approaches). Just as such approaches may be intended to (or may coincidentally) reduce the collateral irAE profile, so too may the liabilities as outlined above be minimized. First in human studies in patients with advanced solid tumors administered pacmilimab in combination with ipilimumab provided evidence that toxicity profiles with this combination were more favorable than standard ICI combinational therapies.^145^

### Clinical Management

The final step in mitigating these toxicities is the acceptance that total avoidance of immunological drug–drug interactions and indeed irAEs overall is not possible. While steps can be taken as outlined above to minimize the impact, mitigation is always subject to risk-benefit, and immunological enhancement as currently clinically applied will always carry some risk. With this duly noted, how patients are managed after identification of irAE or ICI-drug interaction should be optimized as far as possible to minimize toxicity and treatment downtime. As discussed above, irAEs are typically treated with corticosteroids in a standardized fashion which may decrease the clinical efficacy of ICIs.^45^ Therefore, newer management paradigms must be pursued that offer amelioration without losing the antitumor efficacy of ICIs and without the risk of additional ADRs. Alternative medications for the treatment of irAEs that maintain antitumor efficacy when administered in combination with ICIs should be considered and evaluated. Desensitization protocols have the potential to be viable alternative methods for clinical management. Examples of success of desensitization protocols in the clinic are found in the treatment of cystic fibrosis (CF) patients where their medications are essential and life changing. The risk of adverse events outweighs the risk of their CF being left untreated; therefore, desensitization to tazocin and tobramycin has been possible in some cases where delayed type hypersensitivity reactions have occurred.^146^ Similarly, to the treatment of CF patients, the treatment of oncology patients requires careful consideration of the risk-benefit of adverse reactions occurring due to their therapy but also the treatment of their cancer, attempts of desensitization may be the most beneficial for patients when no other viable cancer treatment option is available. A clinical example of a patient with NSCLC with EGFR Thr790Met-mutation who presented with hepatotoxicity caused by osimertinib as their sixth line of therapy was successfully orally desensitized to osimertinib.^147^ Recently, Lopez et al. reported a case where an NSCLC patient suffered an osimertinib-induced SJS reaction after the administration of pembrolizumab (last cycle 2 weeks prior to osimertinib administration); four years later, osimertinib desensitization was successfully carried out with no reoccurrence of SJS after the rechallenge.^102^ This report highlights the potential for patients to tolerate concomitant medications after carefully carried out desensitization protocols and certain time has elapsed after ICI administration.

## Discussion

IO patients represent a cohort of individuals in which polypharmacy is common; this is at least in part due to combinational approaches taken within oncology and the increasing comorbidites of an increasingly complex cancer population.^148,149^ An important question to address is to what extent the drug–drug interactions influence the clinical outcome in terms of safety and efficacy. In light of the burden of clinical evidence summarized herein, it appears that administration of ICI agents may inadvertently push individuals toward an immunological state in which hypersensitivity reactions are more likely and that hypersensitivity to coadministered medications therefore represents a subcomponent of the irAE profile.

Unfortunately, irAEs at present are an inevitable feature across the IO-treated population. The main aim should therefore be to learn from them when they do occur; understanding the cause often leads to understanding optimal clinical management and potentially reduces the need for significant immunosuppression in cases where an extrinsic propagator can be identified. From a drug development perspective, the burgeoning field of IO is likely to offer hundreds of combinations. At present, a major challenge is understanding what makes a combination tolerable (or not). In many respects these challenges reflect those of “conventional” hypersensitivity reactions, but the margin of tolerance seems to be narrowed and often require immunosuppressive treatment strategies once established; however, optimal management strategies remain elusive, as do proactive, prospective concomitant medication strategies aimed at reducing hypersensitivity reactions in this patient cohort.

## Abbreviations

AIN: Acute interstitial nephritis
ADR: Adverse drug reaction
ADA: Antidrug antibody
CTLA-4: Cytotoxic T-lymphocyte-associated protein-4
DILI: Drug-induced liver injury
DRESS: Drug reaction with eosinophilia and systemic symptoms
EGFR: Epidermal growth factor receptor
ICI: Immune checkpoint inhibitor
IO: Immune-oncology
irAEs: Immune-related adverse events
IL: Interleukin
LAG-3: Lymphocyte activation gene-3 protein
LTTs: Lymphocyte transformation tests
mAb: Monoclonal antibody
MHC: Major histocompatibility complexes
SMX-NO: Nitroso-sulfamethoxazole
NSCLC: Non-small-cell lung cancer
PD-1: Programmed death protein 1
PD-L1: Programmed death-ligand 1
PPI: Proton pump inhibitor
SJS: Stevens-Johnson syndrome
Treg: T-regulatory
TEN: Toxic epidermal necrolysis
TME: Tumor microenvironment
TNF-α: Tumor necrosis factor-alpha
TKI: Tyrosine kinase inhibitor
